# Context-Dependent Modulation of Breast Cancer Cell E-Cadherin Expression, Mitogenesis, and Immuno-Sensitivity by Immortalized Human Mesenchymal Stem Cells In Vitro

**DOI:** 10.3390/cells14171316

**Published:** 2025-08-26

**Authors:** Bei Dai, Neha Atale, Amanda M. Clark, Alan Wells

**Affiliations:** 1 Department of Pathology, School of Medicine, University of Pittsburgh, Pittsburgh, PA 15213, USA; daib19@mails.tsinghua.edu.cn (B.D.); amc235@pitt.edu (A.M.C.); 2 School of Medicine, Tsinghua University, Beijing 100084, China; 3 Cell Biology Program, Hillman Cancer Center, University of Pittsburgh, Pittsburgh, PA 15213, USA; 4 R&D Service, Pittsburgh VA Health System, Pittsburgh, PA 15213, USA; 5 Department of Bioengineering, University of Pittsburgh, Pittsburgh, PA 15213, USA

**Keywords:** mesenchymal stem cell (MSC), breast cancer, epithelial–mesenchymal plasticity, TRAIL-induced apoptosis

## Abstract

The major event that leads to death from breast cancer (BrCa) is the emergence of micrometastases into lethal growing metastases. While it is still uncertain what regulates the cell fate decision between remaining in dormancy and aggressive proliferative progression, accumulating evidence demonstrates a major role for the metastatic microenvironment. One area of interest is that of tissue and circulating mesenchymal stem cells (MSCs), which have been shown to alter the proliferative and metastatic potential of BrCa. Herein, we investigate how these cells impact the phenotype of metastatic BrCa. As the disseminated BrCa cells initially adopt an epithelial phenotype in ectopic organs, one that is dormant in having limited proliferation and being immune-silent, interactions that revert the disseminated metastatic BrCa to aggressive mesenchymal phenotypes, would be a driver of metastatic progression. BrCa cells exhibited phenotypic changes including increased E-cadherin expression, altered proliferation, and differential sensitivity to TRAIL-induced apoptosis when directly co-cultured with immortalized human MSCs, compared to the BrCa cells not co-cultured. These regulatory effects were dependent upon the BrCa cell’s epithelial–mesenchymal status and involved distinct juxtacrine and paracrine signaling mechanisms, as evidenced by differing responses in direct co-culture, conditioned medium, and Transwell systems. Our findings highlight the complex and context-dependent roles of MSCs in BrCa progression, improving our understanding of tumor-stroma interactions and laying groundwork for future therapeutic exploration.

## 1. Introduction

Breast cancer (BrCa) remains the most common and deadliest cancer among women, and the second most frequently diagnosed cancer across all genders [1]. Although the incidence of distant-stage BrCa is markedly lower than that of localized or regional disease, its 5-year relative survival rate—approximately 32%—is by far the lowest and accounts for the majority of BrCa deaths [2]. While early-stage tumors are often manageable through surgery, chemotherapy, and radiation, the clinical detection of cancer cells in distant organs significantly reduces therapeutic efficacy and survival outcomes [3]. This is confounded by the knowledge that escape of cells from the primary tumor is not rare, but that the major barrier is survival in the ectopic organ, followed by outgrowth [4,5,6,7].

Increasing evidence suggests that the outcome of dissemination is not solely determined by tumor-intrinsic factors, but is profoundly influenced by the tumor microenvironment (TME). The TME, comprising stromal cells, immune cells, extracellular matrix components, and secreted factors, plays a pivotal role in regulating cancer cell behavior [8]. It can either suppress or promote metastatic outgrowth depending on the dynamic interactions among its components [9]. Of particular interest is the role of mesenchymal stem cells (MSCs), which have been shown to modulate epithelial–mesenchymal plasticity, immune evasion, and resistance to therapy [10,11]. Understanding how the TME contributes to metastatic reactivation is essential for developing targeted strategies to prevent or delay disease recurrence.

MSCs play multifaceted roles in the BrCa TME. MSCs exhibit tropism toward tumor sites via cytokines and chemokines [12,13], inspiring the studies about regulations of MSCs on BrCa cells or MSCs directly act as drug-delivery vehicles [14,15]. MSCs modulate BrCa progression through paracrine signaling, extracellular vesicles, and direct cell interactions. It was both reported that MSCs either promote or suppress tumor growth [16,17,18,19,20,21] and epithelial–mesenchymal plasticity (EMT) [22,23,24,25,26,27,28], depending on cancer type, MSC origin, and microenvironmental cues. Furthermore, MSCs modulate BrCa progression through interacting with other components of TME such as immune cells, macrophages, fibroblasts, and extracellular matrix [29,30]. Clinically, MSCs have been explored as delivery vehicles for anti-tumor agents like Tumor necrosis factor-related apoptosis-inducing ligand (TRAIL) and IFN-β [31,32,33,34,35], some of which have progressed to clinical trials [14,36], yet their effects on therapeutic sensitivity are only partially understood. To develop a unified model that accounts for these diverse reports, we focused on the phenotypic status of the disseminated cells.

E-cadherin, the hallmark epithelial marker, plays a crucial role in regulating tumor cell plasticity and metastatic progression. In the early stages of metastasis, cancer cells often downregulate E-cadherin and weaken cell–cell adhesion, thereby enabling detachment from the primary tumor mass [37]. This loss of adhesion facilitates invasion and dissemination to distant organs. At secondary sites, metastatic colonization often involves a partial mesenchymal-to-epithelial reverting transition (MErT), during which tumor cells re-establish adhesive contacts with neighboring tumor or stromal cells to support survival signaling [38].

These context-dependent transitions help explain the seemingly paradoxical roles of E-cadherin in tumor progression. On one hand, E-cadherin suppresses tumor development through interactions with β-catenin [39] and growth factor receptors such as c-Met [40]. On the other hand, under certain conditions, it may promote tumor growth [41] and enhance therapy resistance [42].

Notably, both clinical data and mouse models have revealed that many metastatic cells exist in a hybrid EMT state, expressing epithelial markers such as E-cadherin while retaining mesenchymal features like vimentin and Fibroblast-specific protein 1 (FSP1) [41,43]. These findings highlight the importance of monitoring E-cadherin not only as a marker of epithelial identity, but also as a dynamic regulator of metastasis. Herein, we report on the effects of MSCs being dependent upon a BrCa cell’s E-cadherin expression status and that it links to both proliferation and immuno-sensitivity of the BrCa.

## 2. Materials and Methods

### 2.1. Cell Lines and Cell Culture

Human BrCa cell lines MDA-MB-231 (M231), MDA-MB-231 with E-cadherin and RFP (231E), MDA-MB-231 with RFP (231R) [38], MDA-MB-468 (M468), MCF-7 (M7), and MCF-7 with E-cadherin knockdown (M7shE) were used. M231, M468, and M7 were purchased from ATCC. 231E were maintained in selection medium with 900 μg/mL G418 and 5 μg/mL puromycin and 231R with 5 μg/mL puromycin. M7shE was generated by transfecting shRNA vectors via Lipofectamine 2000 (Invitrogen, Carlsbad, CA, USA). Details of shRNA sequences and E-cadherin knockdown validation are shown in Supplementary Figure S1. Immortalized human mesenchymal stem cells (ihMSCs) were derived from human bone marrow (gift from Dr. Junya Toguchida, Kyoto University, described in [44]) and characterized by flow cytometry and Oil Red O staining (see Supplementary Figure S2). Cells were CD105^+^, CD90^+^, CD133^−^, CD34^−^, and retained adipogenic differentiation capacity. All cells were cultured in RPMI-1640 supplemented with 10% FBS and 0.5% P/S, with medium changes every 2–3 days.

### 2.2. Co-Culture Experiments

Direct co-culture, indirect Transwell culture, and conditioned medium (CM) treatments were performed. For Transwell culture, 0.4 μm inserts (Corning, Corning, NY, USA) were used. For CM experiments followed by E-cadherin protein detection, CM was collected from mono- or co-cultures of BrCa cells and/or ihMSCs after medium replacement with RPMI-1640 containing 0.5% FBS, 1 mM sodium pyruvate, 1 mM L-glutamine, 1 μM NEAA, and 0.5% P/S at ~70% confluency. Supernatant was collected after 24 h, centrifuged at 3000× *g* for 5 min, supplemented with 10% FBS, and stored at 4 °C. Co-culture conditions are detailed in Supplementary Table S1.

### 2.3. Annexin v/Propidium Iodide (AV/PI) Staining and Flow Cytometry

When reaching ~50% confluency, cells were treated with TRAIL (50 ng/mL for M231 and M7, 5 ng/mL for M468; Thermo Fisher Scientific, Waltham, MA, USA) and cycloheximide (CHX; 5 μM for M231 and M7; 0.5 μM for M468; MilliporeSigma, Burlington, MA, USA) in RPMI-1640 with 0.5% FBS and 0.5% P/S for 24 h, harvested through trypsinization, stained with Fc receptor binding inhibitor (1:100 diluted; Invitrogen, Carlsbad, CA, USA) in 1% FBS/PBS (without Ca^2+^ and Mg^2+^) for 15 min and APC-conjugated anti-CD90 antibody (1:50; BD Biosciences, Cat# 559869, San Jose, CA, USA) for 30 min, and subjected to Pacific Blue-conjugated AV (Invitrogen, Cat# R37177, Carlsbad, CA, USA) and PI (1 μg/mL) staining in annexin V-binding buffer (ABB) for 15 min. ABB was diluted from 10× ABB that composed of 0.1 M HEPES, 1.4 M NaCl, and 24 mM CaCl_2_ in PBS. Flow cytometry was performed immediately. Heat-treated cells (55 °C for 20 min) served as positive controls. Data acquisition and analysis were conducted using BD Canto II flow cytometer (BD Biosciences, San Jose, CA, USA) and FlowJo software (version 10), respectively.

### 2.4. Immunofluorescence (IF)

Cells were fixed in 3.7% formaldehyde for 20 min, blocked in 1% bovine serum albumin (BSA) for 30 min, and incubated with mouse anti-E-cadherin antibody (1:100 in 1% BSA; Thermo Fisher Scientific, Cat# 13-5700, Waltham, MA, USA), overnight at 4 °C or for 1 h at room temperature, followed by Alexa Fluor 488- or Alexa Fluor 594-conjugated goat anti-mouse secondary antibody (1:1000 in 1% BSA; Thermo Fisher Scientific, Cat# A11001 and Cat# A11032, respectively, Waltham, MA, USA) for 1 h, followed by DAPI staining (1 μg/mL; 1 min). No permeabilization was performed. Coverslips were mounted and imaged using fluorescent microscopes.

For baseline characterization of human breast cancer cell lines, cells were maintained as monolayer culture supplemented with RPMI with 10% FBS and 0.5% P/S up to 72 h. Cells were fixed, permeabilized with 0.5% (*w*/*v*) Triton-X 100 for 20 min and blocked. After that cells were stained using anti-Occludin (1:100 in 1% BSA, cell signaling Cat# 91131T) and anti-ZO-1 (Zona Occludens) antibodies (1:100 and anti ZO-1, in 1% BSA, cell signaling Cat# 13663T) for 1 h at room temperature. Alexa Fluor 488-conjugated goat anti-rabbit secondary antibody (1:1000 in 1% BSA; Thermo Fisher Scientific, Cat# A11034, Waltham, MA, USA) was used for detection followed by DAPI staining as described above and imaged using Nikon A1 confocal microscope (Nikon Instruments, Melville, NY, USA).

### 2.5. Western Blot (WB)

Cells were lysed with SDS RIPA buffer composed of 50 mM Tris-HCl (pH 8.0), 150 mM NaCl, 0.1% SDS, 0.5% sodium deoxycholate, 1% NP-40, and 1 mM EDTA (pH 8.0), containing protease inhibitors. Protein concentration was measured using Pierce BCA Protein Assay Kit (Thermo Fisher Scientific, Waltham, MA, USA). Briefly, 10 μL protein lysate was mixed with 100 μL BCA working reagent (made by mixing reagent A and B together in a 49:1 ratio) and incubated at 37 °C for 30 min, followed by reading the optical density value at 560 nm. Equal amounts of protein were mixed with 5× sample buffer (3.9 mL dH_2_O, 1.0 mL 0.5 M Tris pH 6.8, 0.8 mL glycerol, 1.6 mL 10% SDS, 0.4 mL 2-mercaptoethanol, 0.4 mL 1% bromophenol blue), boiled at 100 °C for 5 min, and resolved on 10% SDS–polyacrylamide gels. Proteins were transferred to PVDF membranes using standard wet transfer methods, blocked in 5% milk/TBST for 20 min, and incubated overnight at 4 °C with one of the following primary antibodies diluted in 1% milk/TBST: mouse anti-E-cadherin (1:1500; BD Biosciences, Cat# 610182, San Jose, CA, USA), mouse anti-GFP (1:1000; Santa Cruz Biotechnology, Cat# sc-9996, Dallas, TX, USA), or rabbit anti-GAPDH (1:15,000; Cell Signaling Technology, Cat# 5174S, Danvers, MA, USA). After washing, membranes were incubated for 1 h at room temperature with HRP-conjugated anti-mouse (1:1000; MilliporeSigma, Cat# A4416-1ML, Burlington, MA, USA) or anti-rabbit (1:5000; MilliporeSigma, Cat# A9169-2ML, Burlington, MA, USA) secondary antibodies, also diluted in 1% milk/TBST. Detection was performed using chemiluminescence reagents (Thermo Fisher Scientific, Cat# 32106, Waltham, MA, USA) and X-ray film. Band intensity was quantified with ImageJ (version 1.54d) and normalized to GAPDH.

### 2.6. Reverse Transcription Quantitative Polymerase Chain Reaction (RT-qPCR)

RNA was extracted using TRIzol (Invitrogen, Carlsbad, CA, USA) and reverse transcribed using QuantiTect Reverse Transcription Kit (QIAGEN, Hilden, Germany). qPCR was performed using SYBR Green reagents (Thermo Fisher Scientific, Cat# 4367659, Waltham, MA, USA) in 20 μL reactions containing 10 SYBR Green reagents, 2 µL cDNA template, 4 µL of forward/reverse primer mix, and 4 µL RNase-free water. Thermal cycling was conducted in Agilent Mx3005P (Agilent Technologies, Santa Clara, CA, USA) with an initial denaturation at 95 °C for 10 min, followed by 40 cycles of 95 °C for 15 s and 60 °C for 30 s. Data was analyzed using the 2^−ΔΔ*Ct*^ method, with GAPDH as the endogenous control. Primer sequences were as follows:

hGAPDH-forward (5′→3′): GTCTCCTCTGACTTCAACAGCG
hGAPDH-reverse (5′→3′): ACCACCCTGTTGCTGTAGCCAA
hE-cadherin-forward (5′→3′): GCCTCCTGAAAAGAGAGTGGAAG
hE-cadherin-reverse (5′→3′): TGGCAGTGTCTCTCCAAATCCG

### 2.7. EdU Assay

Twelve hours after seeding cells on coverslips, BrCa cell culture medium was replaced with RPMI-1640 containing 1× insulin-transferrin-selenium supplement (ITS; Thermo Fisher Scientific, Waltham, MA, USA) and 0.5% P/S. After another 12 h, either ihMSCs or BrCa cells (direct co-culture) or one of three types of CM (CM treatment) were added to the same well. After 3 days, 10 µM EdU was added and incubated for 12.5 h (direct co-culture), 16.5 h (M468 with CM treatment), or 24 h (M231 and M7 with CM treatment).

CM was collected from mono- or co-cultures of BrCa cells and/or ihMSCs after medium replacement with RPMI-1640 supplemented with 1× ITS at ~70% confluency. After 24 h, Supernatant was collected, centrifuged at 3000× *g* for 5 min, mixed 1:1 with fresh RPMI-1640 medium (with 1× ITS and 0.5% P/S), and either used immediately or stored at −20 °C for daily replenishment.

EdU detection was performed using the Click-iT Plus EdU Imaging Kit (Thermo Fisher Scientific, Waltham, MA, USA) according to the manufacturer’s protocol. Briefly, coverslips were fixed with 3.7% formaldehyde/PBS for 15 min, permeabilized with 0.5% Triton X-100 (MP Biomedicals, Irvine, CA, USA)/PBS for 15 min, and then incubated with Click-iT Plus reaction cocktail (prepared as per the manufacturer’s instructions) in the dark for 30 min. Between each step, the coverslips were washed twice with 3% BSA/PBS. Coverslips were counterstained with DAPI (1 μg/mL, 3 min), mounted, and imaged on a Nikon A1 confocal microscope (Center for Biologic Imaging, University of Pittsburgh, Pittsburgh, PA, USA). For direct co-culture, cells were blocked in 3% BSA/PBS for 30 min, stained with APC-conjugated anti-CD90 antibody (1:100) for 1 h, then Alexa Fluor 647-conjugated goat anti-mouse antibody (1:1000; Thermo Fisher Scientific, Cat# A21235, Waltham, MA, USA) for 1 h, followed by DAPI staining. Image quantification was performed using ImageJ (direct co-culture) or NIS-Elements (CM treatment).

### 2.8. Statistics

Graphs were generated using GraphPad Prism software (version 10.4.2). Data in bar graphs are presented as mean ± Standard Deviation. Statistical data analyses were performed in pairwise manners against the control with unpaired two-tailed Student’s *t*-test. *p* < 0.05 was considered significant.

## 3. Results

**Figure 1:**
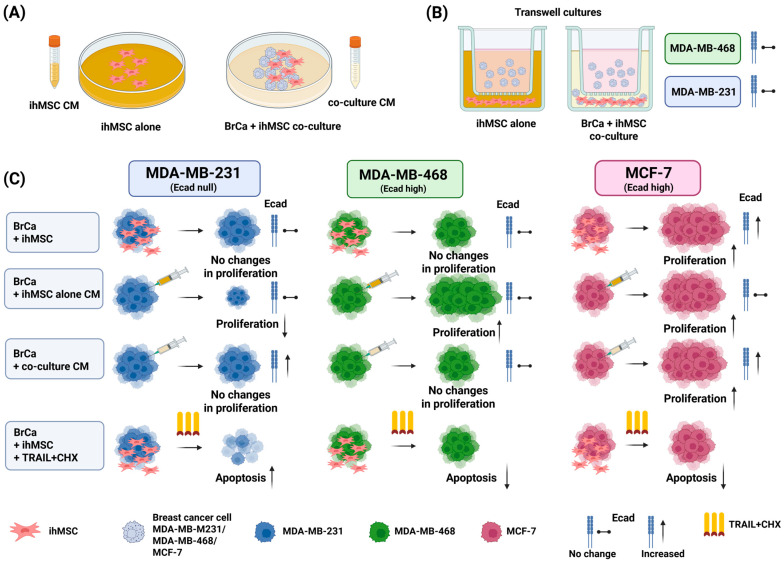
Schematic Overview of the Experimental Workflow and Key Findings. (**A**) Acquirement of two CM types. (**B**) The experimental design and statistical results in Transwell assays. (**C**) The experimental design and statistical results in direct co-culture and CM treatment experiments, followed by analysis of E-cadherin expression, proliferation, and apoptosis proportion (post TRAIL + CHX treatment). Ecad: E-cadherin.

This study involves a number of different in vitro experimental models to decipher the effects of the MSC and whether they need contact, suggesting juxtacrine signaling (which would include cell–cell transfers) or merely paracrine signaling. These models and their outcomes are summarized in Figure 1 for ease.

### 3.1. ihMSCs Enhance E-Cadherin Expression in Breast Cancer Cells

The interaction of MSCs with primary BrCa has been reported to promote both BrCa metastasis [17] and dormancy [45] in vivo. These seemingly mutually exclusive behaviors could be explained by alterations in the epithelial–mesenchymal phenotype switching that occurs during BrCa progression, and thus would have differential effects depending on the stage of progression impacted, similar to the divergent effects of macrophages on BrCa phenotypic shifting [46].

**Figure 2:**
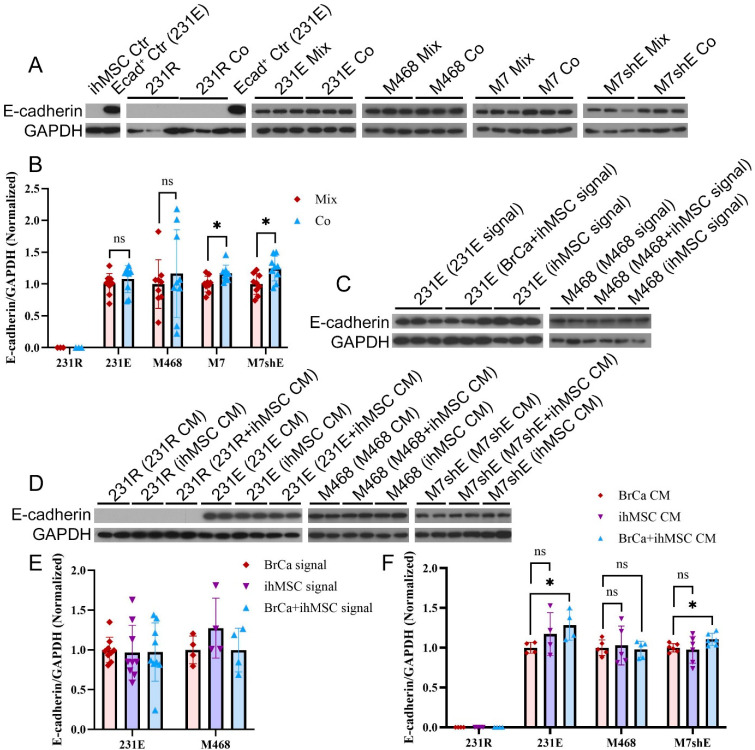
ihMSCs Enhance E-Cadherin Expression in Breast Cancer Cells. (**A**) Representative WB analysis of E-cadherin and GAPDH expression in 231R, 231E, M468, M7, and M7shE cells cultured alone or directly co-cultured with ihMSCs. GAPDH was used as a loading control. Ecad^+^ Ctr: E-cadherin-positive control (Ctr). (**B**) Quantification of protein levels from A, combined with additional independent replicates and normalized to GAPDH and the corresponding control groups (“231R” for 231R, “BrCa Mix” for the other BrCa cell lines). 231R cells (n = 1) and 231E, M468, M7, and M7shE cells (n = 3). (**C**) Representative WB analysis of E-cadherin and GAPDH expression in 231E and M468 cells exposed to BrCa signals, ihMSC signals, or BrCa + ihMSC signals in Transwell assays. GAPDH was used as a loading control. (**D**) Representative WB analysis of E-cadherin and GAPDH expression in 231R, 231E, M468, and M7shE cells treated with BrCa CM, ihMSC CM, or BrCa + ihMSC CM in the CM experiments. GAPDH was used as a loading control. (**E**) Quantification of protein levels from (**C**), combined with additional independent replicates and normalized to GAPDH and the Ctr groups (BrCa signal) (n = 2–3). No statistically significant differences were observed. (**F**) Quantification of protein levels from (**D**), combined with additional independent replicates and normalized to GAPDH and the Ctr groups (BrCa CM). (n = 2–3). ns = not significant. * *p* < 0.05.

We first investigated the effect of MSCs on both E-cadherin-positive and -negative BrCa cell lines. We have found previously that MErT in cancer involves increased epithelial marker expression (connexin 26/43 and E-cadherin) alongside persistent mesenchymal markers (vimentin and FSP1), revealing that phenotypic switching is not just plastic but often incomplete [43]. Therefore, we examined just E-cadherin, as it is the epithelial cell marker responsible for cell–cell cohesion and survival signaling during dormancy [38,43]. To explore whether the E-cadherin levels of BrCa cells are changed by direct contact with ihMSCs, BrCa cells and ihMSCs were co-cultured for up to 4 days. The level of E-cadherin was assessed by immunoblotting, cells not co-cultured being controlled by mixing cell equivalent lysates. The E-cadherin levels of M7 and M7shE cells were upregulated after direct co-culture with ihMSCs, while those of 231R, 231E, and M468 cells remained unchanged (Figure 2A,B). Both of the E-cadherin-modulated cell lines were used here to enhance the visibility of E-cadherin changes. Transwell culture allows for bi-directional communication between the BrCa cells whose phenotype will be detected in the end and the BrCa cells, ihMSCs, or the co-culture system seeded in the other chamber. Cells were maintained as monolayer cultures to minimize the differentiating effects induced by the polarized environment of Transwell cultures. However, to supplement our analysis for baseline characteristics across different breast cancer cell lines, we also stained for other cell–cell cohesion molecules. Occludin and ZO-1 tight junction proteins immunofluorescence staining showed the clear distinction between epithelial and mesenchymal phenotypes. M7 and M468 cells exhibited robust expressions of both proteins, and strong intercellular adhesion. In contrast, these markers were undetectable in the more aggressive metastatic cell lines, including M231, 231R, 231E, and M7shE (see Supplementary Figure S3), suggesting a complete loss of tight junction structures. No changes in E-cadherin expression of the 231E and M468 cells were detected when communicating with ihMSCs or the co-culture system through the Transwell inserts (Figure 2C,E). CM collected from the co-culture of ihMSCs with either 231E or M7shE increased E-cadherin levels in the corresponding BrCa cells, whereas CM from ihMSCs alone did not (Figure 2D,F), suggesting a cooperative role in paracrine signaling.

To validate the immunoblotting results, several complementary experiments were performed. No changes were observed in E-cadherin mRNA levels of 231E cells after direct co-culture with ihMSCs (Supplementary Figure S4A). Morphological analysis showed no apparent epithelial–mesenchymal phenotypic changes in M231, M468, or M7 cells (Supplementary Figure S4B). IF staining confirmed the lack of obvious E-cadherin changes in 231E cells under direct co-culture with ihMSCs (Supplementary Figure S4C). These results were consistent with the immunoblotting data, supporting the specificity and robustness of our findings.

### 3.2. ihMSCs Modulate the Proliferation of Breast Cancer Cells

**Figure 3:**
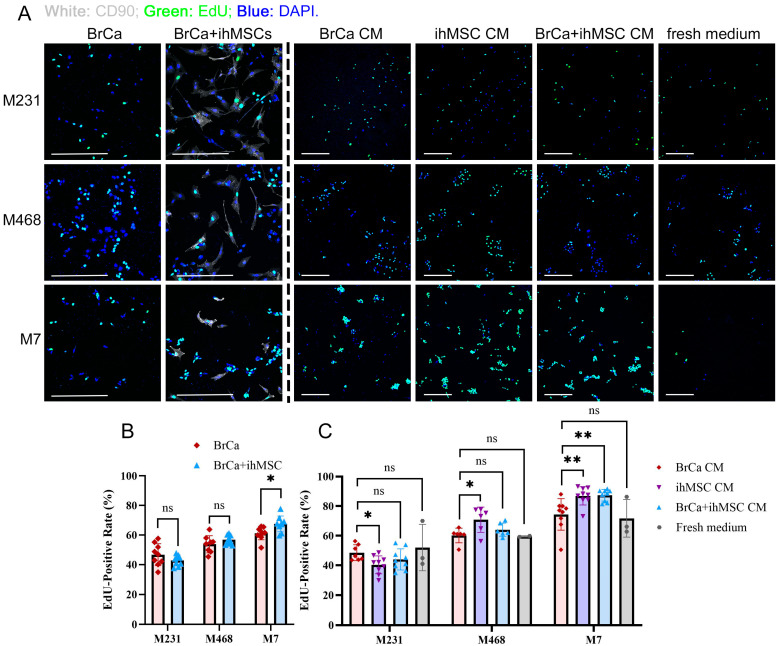
ihMSCs Modulate the Proliferation of Breast Cancer Cells. (**A**) Representative images of EdU incorporation and DAPI staining in M231, M468, and M7 cells under direct co-culture with ihMSCs or treatment with BrCa CM, ihMSC CM, BrCa + ihMSC CM, or fresh RPMI-1640 medium supplemented with 1× ITS and 0.5% P/S. CD90 was stained as ihMSC marker in the direct co-cultures. Images were acquired using a Nikon A1 confocal microscope with total magnifications of 200× (for direct co-culture) or 100× (for CM treatment). White scale bar = 300 μm. (**B**) Quantification of EdU-positive rates in BrCa cells from the direct co-culture groups shown in (**A**), combined with additional independent replicates (n = 3). (**C**) Quantification of EdU-positive rates in BrCa cells from the CM or fresh medium treatment groups shown in (**A**), combined with additional independent replicates (n = 2–3) ns = not significant. * *p* < 0.05, ** *p* < 0.01.

One key difference in the mesenchymal phenotype is greater proliferation. Direct co-culture also regulates BrCa cell proliferation. Detected by EdU staining, M7 cells grew faster when directly co-cultured with ihMSCs, while the proliferation rates of M231 and M468 cells were not influenced (Figure 3A,B). CM derived from ihMSCs promoted the proliferation of both M468 and M7 cells but suppressed that of M231 (Figure 3A,C). This regulatory effect on M7 cells was preserved when CM was collected from co-cultures of ihMSCs and BrCa cells. In contrast, direct co-culture or co-culture-derived CM did not alter the proliferation of M231 and M468 cells, suggesting that direct contact may suppress the production or action of these soluble factors. To reflect the overall trend in proliferation changes, Table 1 presents aggregated EdU-positive rates of BrCa cells following ihMSC direct co-culture or exposure to various types of CM.

### 3.3. ihMSCs Influence the Sensitivity of Breast Cancer Cells to TRAIL-Induced Apoptosis

**Figure 4:**
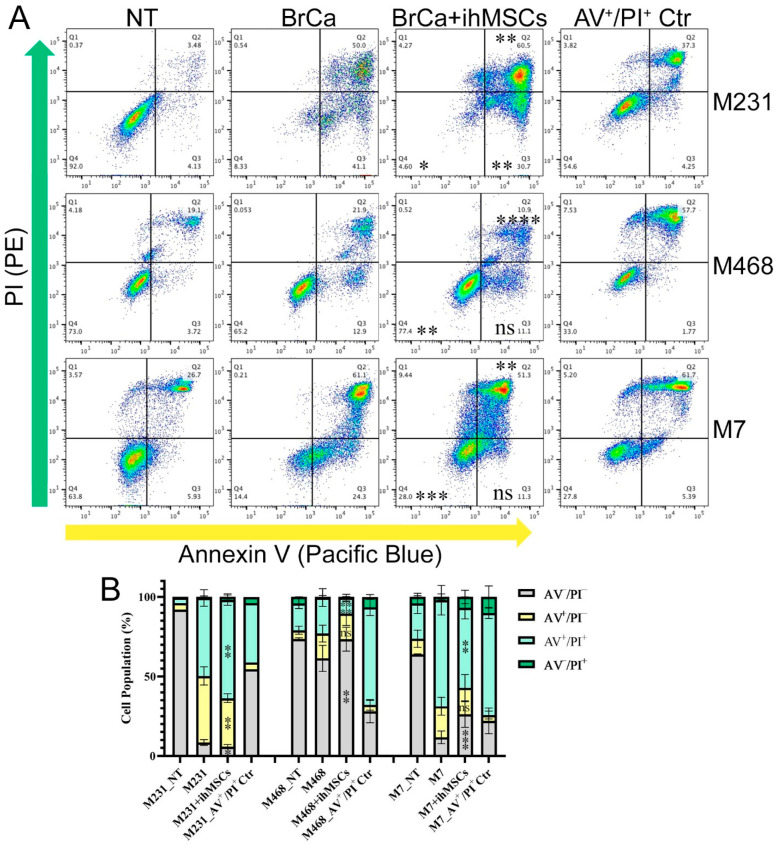
ihMSCs Influence the Sensitivity of Breast Cancer Cells to TRAIL-Induced Apoptosis. (**A**) Representative flow cytometry profiles of AV (Pacific Blue channel) and PI (PE channel) double staining in M231, M468, and M7 cells treated with TRAIL and CHX. Statistical comparisons were performed relative to the BrCa group. (**B**) Quantitative analysis of cell populations shown in (**A**) (n = 2–3). Statistical comparisons were performed relative to the BrCa group. NT: non-treatment. ns = not significant. * *p* < 0.05, ** *p* < 0.01, *** *p* < 0.001, **** *p* < 0.0001.

TRAIL and Fas ligand (FasL) are apoptosis inducers expressed ubiquitously inside and outside the immune system. They broadly participate in innate immunity to kill invading cells and thus the initial clearance of cancer cells. As BrCa cells showed low sensitivity to FasL (Supplementary Figure S5), BrCa cells were treated with TRAIL-CHX to mimic the resistance of disseminated cells to the initial foreign body immune response that occurs upon seeding of ectopic organs. CHX, a protein synthesis inhibitor, was added to mimic the cell starvation that is common during tumor growth. Once treated with TRAIL-CHX, the BrCa cell population move from the live cell area (AV^−^/PI^−^ area) to the early apoptosis (AV^+^/PI^−^) area and the late apoptosis (AV^+^/PI^+^) area. But the pre-co-culture with ihMSCs partially prevented this transition in M7 and M468, implying the protection effect of ihMSCs (Figure 4). However, this effect becomes the promotion of apoptosis in M231. Similarly with the impact of ihMSCs on BrCa cell proliferation, the regulation direction is likely determined by the mesenchymal–epithelial status of the BrCa cells in which M231 cells are E-cadherin negative while M7 and M468 cells are E-cadherin positive.

## 4. Discussion

Our study demonstrates that ihMSCs exert complex and cell context-specific regulatory effects on BrCa cells, particularly in modulating E-cadherin expression, cell proliferation, and resistance to TRAIL-induced apoptosis—three key behaviors that contribute to metastatic seeding. These effects are context-dependent and influenced by the epithelial–mesenchymal phenotype of the cancer cells. By comparing direct co-culture, Transwell assays, and CM exposure, we reveal distinct roles of paracrine and juxtacrine signaling in mediating these interactions. Collectively, our findings suggest that MSCs have a dual, and sometimes seemingly contradictory, influence on BrCa progression.

E-cadherin is a key epithelial marker that maintains cell–cell adhesion and plays a critical role in regulating tumor dormancy and metastasis. In addition to the findings that BrCa cells display expression infidelity in terms of phenotypic markers [43], we chose to just detect E-cadherin as shorthand for the epithelial phenotype, as the other markers such as N-cadherin and vimentin do not faithfully track with cell–cell cohesion in cancer cells. Further, E-cadherin provides for the possibility of complete cell–cell cohesion and communication and thus is the foundational change for the epithelial phenotype [47]. Our data shows that direct contact with ihMSCs promotes E-cadherin expression in M7shE and M7 cells. This up-regulation was also observed in the M7shE cells exposed to “BrCa cell-ihMSC co-culture” CM rather than “ihMSC” CM, suggesting the direct contact between BrCa cells and ihMSCs is required. Interestingly, although the direct contact with ihMSCs has no effect on E-cadherin expression of 231E cells, a similar upregulation was still observed in the 231E cells exposed to the “co-culture” CM but not the “ihMSC” CM. This may be due to the differences between juxtacrine signals and paracrine signals: the upregulation of E-cadherin levels appears to depend on paracrine factors secreted by ihMSCs in response to signals from BrCa cells, or vice versa, whereas juxtacrine signals from direct contact with ihMSCs may counteract or inhibit this effect. This duality may contribute to the seemingly contradictory roles MSCs play in cancer: supporting both dormancy [45] and metastasis [17] depending on the timing and location of interaction during tumor progression. Apart from that, no regulation was observed in Transwell co-cultures, which may be attributed to the lack of direct cell–cell contact and/or insufficient accumulation of secreted factors in contrast to the CM experiments. These observations underscore the nuanced role of ihMSCs in epithelial–mesenchymal plasticity and highlight the importance of direct cell–cell interaction in this regulation.

In terms of cell proliferation, we found that exposure to ihMSC CM significantly enhanced the proliferation of both M468 and M7 cells, while suppressing the proliferation of M231 cells. This differential effect may depend on the intrinsic epithelial–mesenchymal phenotype of cancer cells. M231 cells, being more mesenchymal, may respond to MSC-derived signals by entering a growth-suppressive or quiescent state, while more epithelial-like cells such as M7 and M468 exhibit enhanced proliferation. Additionally, it appears consistent that among the three BrCa cell lines, only M7 cells were affected by the “BrCa cell-ihMSC co-culture” CM, and similarly, only M7 cells responded to direct co-culture with ihMSCs. This suggests that cell–cell contact may interfere with or counteract the effect of ihMSC-secreted factors on the proliferation of M231 and M468 cells, whereas the effect on M7 cells is not subject to this limitation. Breast cancer cells exhibiting a migratory phenotype typically display reduced cell–cell adhesion and enhanced matrix-degrading capabilities. In addition to the E-cadherin differences, the distribution of the tight junction proteins Occludin and ZO-1, were higher in M7 and M468 cells revealed the epithelial characteristics, however loss of these markers was observed in all of M231 mesenchymal cell types, clearly providing a baseline profile of the epithelial–mesenchymal traits of these cells. The absence of these junctional proteins in metastatic cells aligns with the notion that disruption of epithelial integrity facilitates the acquisition of migratory and invasive capabilities. Similarly with the regulations on E-cadherin, these findings suggest that juxtacrine and paracrine signals may have antagonistic roles in modulating tumor cell proliferation, and that the balance between these signals determines the net outcome.

We also examined how ihMSCs influence the apoptotic sensitivity of BrCa cells using TRAIL-CHX treatment, a model that mimics immune clearance during early tumor formation. Pre-exposure to ihMSCs conferred resistance to TRAIL-induced apoptosis in M7 and M468 cells but sensitized M231 cells to cell death. These results mirror the proliferation data and further support the hypothesis that MSCs modulate tumor behavior differently depending on the mesenchymal–epithelial status of the target cells. MSC-mediated protection against apoptosis has been reported in other cancer types and is often attributed to the secretion of anti-apoptotic cytokines such as insulin-like growth factor 1 (IGF1) and leptin [48], or via exosome-mediated delivery of survival signals such as miR-410 [49], miR-21 and miR-34a [50]. Other MSC-secreted anti-apoptotic or pro-survival factors potentially involved in this regulation include vascular endothelial growth factor (VEGF), fibroblast growth factor-2 (FGF-2), platelet-derived growth factor (PDGF), hepatocyte growth factor (HGF), brain-derived neurotropic factor (BDNF), stromal cell-derived factor (SDF)-1α, IGF-2, transforming growth factor-β (TGF-β), IGF binding protein-2 (IGFBP-2) (reviewed in [51]), JAK2 (Janus kinase 2)/STAT3 (signal transducer and activator of transcription 3) pathway, B-cell lymphoma (BCL)-2, BCL-2-associated X protein (BAX) [52,53]. In addition, MSC-derived exosomal miR-381-3p and miR-941 have been reported to induce MErT in BrCa cells [27,28,54]; while miR-410 and miR-1228 were shown to regulate tumor growth [49,55]. Recent studies also indicated that MSCs may exert effects on BrCa cells through mitochondrial transfer [56,57,58]. This mode of cell–cell communication was not explored herein, and lies outside the scope of the current investigation; however it would be considered a possibility for those situations that require MSC coculture, and will be part of future studies. Whether these mechanisms are involved in our system remains to be elucidated.

Taken together, our findings suggest that ihMSCs do not exert a uniform effect on BrCa cells but instead act in a cell-context-specific manner that is heavily influenced by the epithelial–mesenchymal phenotype. This dual functionality may help explain the conflicting roles of MSCs reported in the literature, where they have been shown to promote both tumor suppression and progression [29,59,60,61,62]. Importantly, our study emphasizes the significance of experimental context, particularly the type of intercellular communication involved (direct contact vs. soluble factors), in shaping the outcome of MSC-tumor interactions.

There are several limitations to our study. First, although we used well-characterized cell lines, they may not fully capture the heterogeneity and plasticity of primary breast tumors. ihMSCs could demonstrate differential functional properties compared with primary MSCs, including altered extracellular vesicle release and mitochondrial donation. To enhance generalizability, it would be beneficial to validate our findings using primary MSCs and in vivo models that more accurately recapitulate the TME, including the presence of immune cells, fibroblasts, and extracellular matrix components. Second, we focused primarily on E-cadherin as a phenotypic marker based on prior work demonstrating its over-riding phenotypic contributions [43], but additional markers (e.g., N-cadherin, vimentin) and functional assays (e.g., migration, invasion, or Seahorse metabolic analysis) might provide a more comprehensive picture of epithelial–mesenchymal transition dynamics. Third, while we conclude these effects depend on cancer subtype (M231 and M468 being TNBC and M7 is luminal), we cannot exclude potential confounding effects due to the small number of cell lines examined and restricted experimental approaches. Fourth, while our data suggest various regulations of ihMSCs on BrCa cells, we did not explore the underlying mechanisms. Future studies could investigate the candidate signal factors mentioned in related studies. Upon identification of key regulatory pathways or miRNAs through molecular screening and animal models, corresponding synthetic mimetics, inhibitors, and engineered MSCs could be developed to synergistically enhance anti-tumor effects while suppressing pro-tumor effects, with specificity for the epithelial–mesenchymal phenotype of tumors. These MSC-mediated regulatory mechanisms may further inform the development of optimized MSC-based drug delivery systems, which will be pursued in future studies.

## 5. Conclusions

In conclusion, ihMSCs exert multifaceted effects on BrCa cells by modulating E-cadherin expression, proliferation, and apoptosis sensitivity through both paracrine and juxtacrine mechanisms. These effects are strongly influenced by the phenotypic state of the cancer cells, highlighting the importance of cellular context in interpreting stromal-tumor interactions. Our results contribute to a more nuanced understanding of the MSC–cancer cell relationship and underscore the need to consider both cell-intrinsic properties and intercellular communication modes in future studies and therapeutic strategies.

## Figures and Tables

**Table 1:** Aggregated EdU Proliferation Data of BrCa Cells under ihMSC Direct Co-culture or CM Treatment.

Co-Culture Type	Cell Line	Group	EdU^+^ Nuclei	EdU^−^ Nuclei	Total Nuclei	EdU^+^/Total Nuclei (%)	*p* Value Versus BrCa
Direct	M231	BrCa	714	834	1548	46.12	/
		BrCa + ihMSC	562	734	1296	43.36	0.183
	M468	BrCa	2423	2128	4551	53.24	/
		BrCa + ihMSC	2015	1524	3539	56.94	0.187
	M7	BrCa	1423	874	2297	61.95	/
		BrCa + ihMSC	2137	1060	3197	66.84	0.014
CM	M231	BrCa CM	1388	1431	2819	49.24	/
		ihMSC CM	861	1213	2074	41.51	0.013
		BrCa + ihMSC CM	1099	1273	2372	46.33	0.182
		Fresh medium	255	273	528	48.30	0.600
	M468	BrCa CM	2795	1807	4602	60.73	/
		ihMSC CM	3405	1374	4779	71.25	0.025
		BrCa + ihMSC CM	3865	2169	6034	64.05	0.188
		Fresh medium	793	539	1332	59.53	0.873
	M7	BrCa CM	2512	900	3412	73.62	/
		ihMSC CM	8046	1271	9317	86.36	0.007
		BrCa + ihMSC CM	6761	1079	7840	86.24	0.003
		Fresh medium	791	323	1114	71.01	0.724

Note: *p* values were calculated using unpaired two-tailed Student’s *t*-tests, with each coverslip representing an independent data point. All other values represent aggregated counts from multiple independent experiments.

## Supplementary Materials

The following supporting information can be downloaded at: https://www.mdpi.com/article/10.3390/cells14171316/s1. Figure S1: Establishment and validation of E-cadherin knockdown in M7shE cells; Figure S2: Characterization of ihMSCs; Figure S3: Localization of Occludin and ZO-1 in different breast cancer cells using immunofluorescence staining; Figure S4: Validation of immunoblotting results by RT-qPCR, morphological analysis, and immunofluorescence; Figure S5: Sensitivity of BrCa Cells and ihMSCs to FasL/TRAIL-Induced Apoptosis; Figure S6: Uncropped WB images; Table S1: Conditions for Direct and Indirect Co-cultures.

## Date Availability Statement

All data are contained within the paper and Supplementary Materials.

## Abbreviations

The following abbreviations are used in this manuscript:

ABB	Annexin V-binding buffer
APC	Allophycocyanin
AV	Annexin V
BAX	BCL-2-associated X protein
BCL	B-cell lymphoma
BDNF	Brain-derived neurotrophic factor
BrCa	Breast cancer
BSA	Bovine serum albumin
cDNA	Complementary deoxyribonucleic acid
CFSE	Carboxyfluorescein succinimidyl ester
CHX	Cycloheximide
CM	Conditioned medium
Ctr	Control
DAPI	4′,6-Diamidino-2-phenylindole
DMEM	Dulbecco’s Modified Eagle Medium
DTCs	Disseminated tumor cells
Ecad	E-cadherin
EDTA	Ethylenediaminetetraacetic acid
EdU	5-ethynyl-2′-deoxyuridine
EGFP	Enhanced green fluorescent protein
EMT	Epithelial–mesenchymal transition
FasL	Fas ligand
FBS	Fetal bovine serum
FGF	Fibroblast growth factor
FITC	Fluorescein 5-isothiocyanate
FSP1	Fibroblast-specific protein 1
GAPDH	Glyceraldehyde-3-phosphate dehydrogenase
GFP	Green fluorescent protein
HEPES	4-(2-Hydroxyethyl)-1-piperazineethanesulfonic acid
HGF	Hepatocyte growth factor
HRP	Horseradish peroxidase
IF	Immunofluorescence
IFN	Interferon
IGF1	Insulin-like growth factor 1
IGFBP-2	Insulin-like growth factor-binding protein 2
ihMSCs	immortalized human mesenchymal stem cells
ITS	Insulin-Transferrin-Selenium
JAK2	Janus kinase 2
M231	MDA-MB-231
M468	MDA-MB-468
M7	MCF-7
M7shE	MCF-7 with shRNA-mediated E-cadherin knockdown
MErT	Mesenchymal-to-epithelial reverting transition
miRNA	microRNA
mRNA	Messenger RNA
MSCs	Mesenchymal stem cells
NEAA	Non-essential amino acids
NP-40	Nonidet P-40
NT	Non-treatment
P/S	Penicillin-Streptomycin
PBS−−	Calcium- and magnesium-free phosphate-buffered saline
PBS	Phosphate-buffered saline
PDGF	Platelet-derived growth factor
PE	Phycoerythrin
PI	Propidium iodide
PVDF	Polyvinylidene fluoride
RFP	Red fluorescent protein
RIPA	Radioimmunoprecipitation assay
RNA	Ribonucleic acid
RPMI	Roswell Park Memorial Institute
RT-qPCR	Reverse transcription quantitative polymerase chain reaction
SDS	Sodium dodecyl sulfate
SDF	Stromal cell-derived factor
shRNA	Short hairpin RNA
STAT3	Signal transducer and activator of transcription 3
TBST	Tris-buffered saline with Tween 20
TGF	Transforming growth factor
TME	Tumor microenvironment
TRAIL	Tumor necrosis factor-related apoptosis-inducing ligand
VEGF	Vascular endothelial growth factor
WB	Western blot
231E	MDA-MB-231 with E-cadherin and RFP
231R	MDA-MB-231 with RFP only
ZO-1	Zona Occludens
