# Supplementary material for: Context-Dependent Modulation of Breast Cancer Cell E-Cadherin Expression, Mitogenesis, and Immuno-Sensitivity by Immortalized Human Mesenchymal Stem Cells In Vitro
Source: Cells. 2025 Aug 26;14(17):1316. doi: 10.3390/cells14171316 (PMC12428074; doi:10.3390/cells14171316)
Supplement: Supplementary file 1 [file cells-14-01316-s001.zip › cells-3780817-supplementary.pdf]

Context-Dependent Modulation of Breast Cancer Cell E-Cadherin Expression, Mitogenesis, and Immuno-Sensitivity by Immortalized Human Mesenchymal Stem Cells In Vitro

Supplemental Data

Supplementary Table S1 Conditions for Direct and Indirect Co-cultures

Assessed Parameter	Co-culture Type	Cell Line	Seeding Density (M = Million)	Plate Type	Treatment Duration
E-cadherin protein (WB)	Direct	231R	**ihMSC Ctr:** 0.06M ihMSCs; **Ctr:** 0.14 M 231R; **Co:** 0.07 M 231R + 0.07 M ihMSCs.	6-well plate	4 d
E-cadherin protein (WB)	Direct	231E	**Ctr (Mix):** 0.01 M 231E & 0.01 M ihMSCs; cultured separately, mixed post-lysis; **Co:** 0.005 M 231E + 0.005 M ihMSCs.	6-well plate	4 d
E-cadherin protein (WB)	Direct	M468	**Mix (Ctr):** 0.02 M M468 & 0.02 M ihMSCs; **Co:** 0.02 M M468 + 0.02 M ihMSCs.	6-well plate	4 d
E-cadherin protein (WB)	Direct	M7	**Mix (Ctr):** 0.02 M M7 & 0.02 M ihMSCs; **Co:** 0.02 M M7 + 0.02 M ihMSC.	6-well plate	4 d
E-cadherin protein (WB)	Direct	M7shE	**Mix (Ctr):** 0.01 M M7shE & 0.01 M ihMSCs; **Co:** 0.01 M M7shE + 0.01 M ihMSCs.	6-well plate	4 d
E-cadherin protein (WB)	Transwell	231E	0.044 M 231E in inserts; **231E signal:** 0.1 M 231E in plates; **ihMSC signal:** 0.1 M ihMSCs in plates; **231E+ihMSC signal:** 0.05 M 231E + 0.05 M ihMSCs in plates.	6-well plate	5–7 d
E-cadherin protein (WB)	Transwell	M468	0.04 M M468 in inserts; **M468 signal:** 0.08 M M468 in plates; **ihMSC signal:** 0.04 M ihMSCs in plates; **M468+ihMSC signal:** 0.04 M 231E + 0.04 M ihMSCs in plates.	6-well plate	6 d
E-cadherin protein (WB)	CM	231R	**CM plate:** 0.14 M 231R (treated in 231R CM, ihMSC CM, or 231R+ihMSC CM).	6-well plate	4 d
E-cadherin protein (WB)	CM	231E	**CM plate:** 0.1 M 231E (treated in 231E CM, ihMSC CM, or 231E+ihMSC CM).	6-well plate	4 d
E-cadherin protein (WB)	CM	M468	**CM plate:** 0.15 M M468 (treated in M468 CM, ihMSC CM, or M468+ihMSC CM).	6-well plate	4 d
E-cadherin protein (WB)	CM	M7shE	**CM plate:** 0.3 M M7shE (treated in M7shE CM, ihMSC CM, or M7shE+ihMSC CM).	6-well plate	4 d
E-cadherin mRNA	Direct	231E	**Mix (Ctr):** 0.1 M 231E & 0.1 M ihMSCs; **Co:** 0.05 M 231E + 0.05 M ihMSCs.	6-well plate	4 d
Morphology	Direct	M231	**Ctr:** 0.0075 M M231; **Co:** 0.0075M M231 + 0.0075 M ihMSCs.	12-well plate	4 d
Morphology	Direct	M468	**Ctr:** 0.01 M M468; **Co:** 0.01 M M468 + 0.01 M ihMSCs.	12-well plate	4 d
Morphology	Direct	M7	**Ctr:** 0.01 M M7; **Co:** 0.01 M M7 + 0.01 M ihMSCs.	12-well plate	4 d
E-cadherin protein (IF)	Direct	231E	**Ctr:** 0.08 M 231E; **Co:** 0.04 M 231E + 0.04 M ihMSCs.	6-well plate	3 d
EdU	Direct	M231	**ihMSC Ctr:** 0.015 M ihMSCs **Ctr:** 0.01 M M231 (+ 0.01 M M231 seeded later) **Co:** 0.01 M M231 (+ 0.01 M ihMSCs seeded later)	12-well plate	3 d
EdU	Direct	M468	**ihMSC Ctr:** 0.015 M ihMSCs **Ctr:** 0.01 M M468 (+ 0.01 M M468 seeded later) **Co:** 0.01 M M468 (+ 0.01 M ihMSCs seeded later)	12-well plate	3 d

EdU	Direct	M7	**ihMSC Ctr:** 0.015 M ihMSCs<br>**Ctr:** 0.01 M M7 (+ 0.01 M M7 seeded later)<br>**Co:** 0.01 M M7 (+ 0.01 M ihMSCs seeded later)	12-well plate	3 d
EdU	CM	M231	**CM plate:** 0.02 M M231 (treated in M231 CM, ihMSC CM, M231+ihMSC CM, or fresh medium).	12-well plate	3 d
EdU	CM	M468	**CM plate:** 0.02 M M468 (treated in M468 CM, ihMSC CM, M468+ihMSC CM, or fresh medium).	12-well plate	3 d
EdU	CM	M7	**CM plate:** 0.02 M M7 (treated in M7 CM, ihMSC CM, M7+ihMSC CM, or fresh medium).	12-well plate	3 d
AV/PI	Direct	M231	**Ctr:** 0.0075 M M231;<br>**Co:** 0.0075 M M231 + 0.0075 M ihMSCs.	12-well plate	4−6 d
AV/PI	Direct	M468	**Ctr:** 0.01 M M468;<br>**Co:** 0.01 M M468 + 0.01 M ihMSCs.	12-well plate	4−6 d
AV/PI	Direct	M7	**Ctr:** 0.01 M M7;<br>**Co:** 0.01 M M7 + 0.01 M ihMSCs.	12-well plate	4−6 d

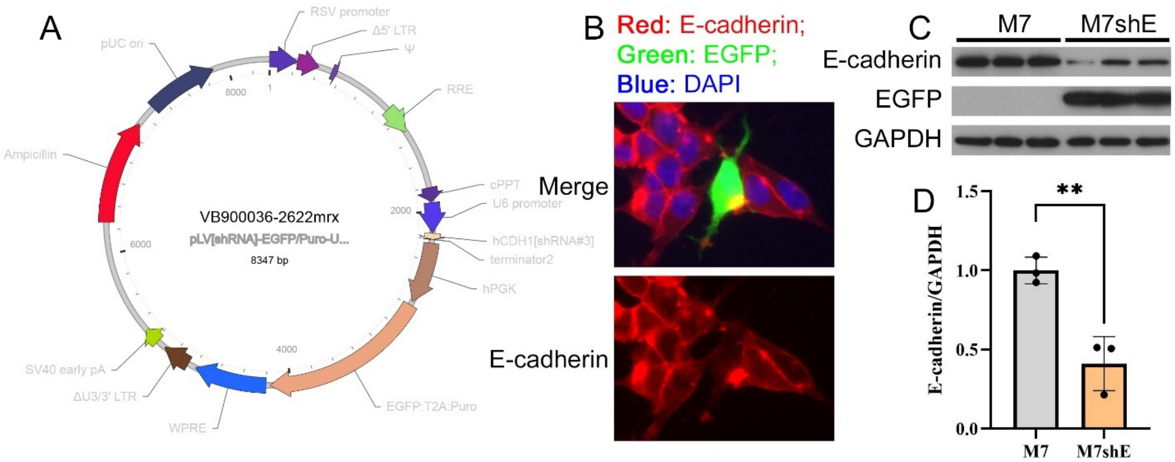

Supplementary Figure S1. Establishment and validation of E-cadherin knockdown in M7shE cells. **(A)** Schematic of the E-cadherin shRNA vector used to generate M7shE cells. The image was obtained from the VectorBuilder website (https://en.vectorbuilder.com/vector/VB900036-2622mrx.html). Targeting shRNA sequence: 5'-TTTCGGCAGTTCAAGCTATATCTCGAGATATAGCTTGAACTGCCGAAA-3'. Copyright belongs to the original source. **(B)** Representative IF images of M7shE cells prior to stable selection. EGFP served as a marker of shRNA expression. Image acquired at 200× magnification under the Olympus IX70 inverted fluorescence microscope; cropped regions (1/6 of the original image width) are shown. **(C)** Representative immunoblot analysis of E-cadherin, EGFP, and GAPDH expression in parental M7 and M7shE cells after stable selection. EGFP indicates shRNA expression; GAPDH serves as a loading control. **(D)** Densitometric quantification of protein levels shown in (C), normalized to GAPDH. ** $p < 0.01$.

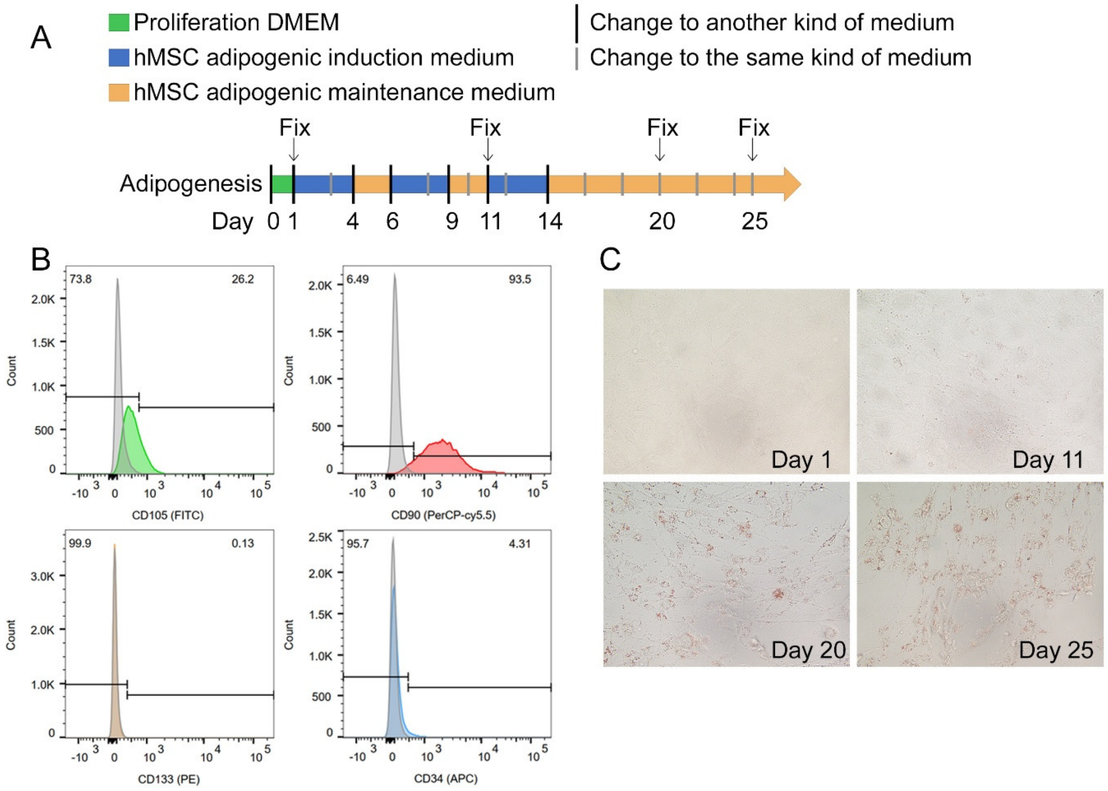

Supplementary Figure S2. Characterization of ihMSCs **(A)** Procedure of adipogenic differentiation induction. The human MSC adipogenic differentiation medium bulletkit (Lonza, Cat# PT-3004) was used. Proliferation DMEM**:** DMEM with 10% FBS, 1 mM sodium pyruvate, 1 mM L-glutamine, 1 μM NEAA, and 100 units/mL P/S. **(B)** Flow cytometry profiles of CD105, CD90, CD133, and CD34 expressions in ihMSCs. Cells were digested with pre-warmed enzyme-free cell dissociation buffer (Thermo Fisher Scientific), fixed in 1% paraformaldehyde/PBS for 20 min, resuspended in 1% FBS/PBS, and incubated with Fc receptor binding inhibitor for 15 min followed by staining with one of the following conjugated antibodies for 30 min at room temperature: PerCP-Cyanine5.5 anti–CD90 (1:1000; Thermo Fisher Scientific, Cat# 45-0909-41), APC anti–CD34 (1:20, Thermo Fisher Scientific, Cat# 17-0349-41), PE anti–CD133 (1:100; Thermo Fisher Scientific, Cat# 12-1338-41), or FITC anti–CD105 (1:100; BD Biosciences, Cat# 561443). Compensation was performed using UltraComp eBeads Plus Compensation Beads (Thermo Fisher Scientific). Acquisition and analysis were conducted using BD FACSDiva and FlowJo software. **(C)** The Oil Red O staining result of ihMSCs after the time-gradient adipogenic induction that started on day 1. Adipogenesis-induced ihMSCs were fixed, pretreated with 60% isopropanol for 5 min, and stained with Oil Red O for 15 min. Coverslips were rinsed with 60% isopropanol, washed, mounted, and imaged on an Olympus IX70 inverted microscope using 400× magnify. Red: fat droplets.

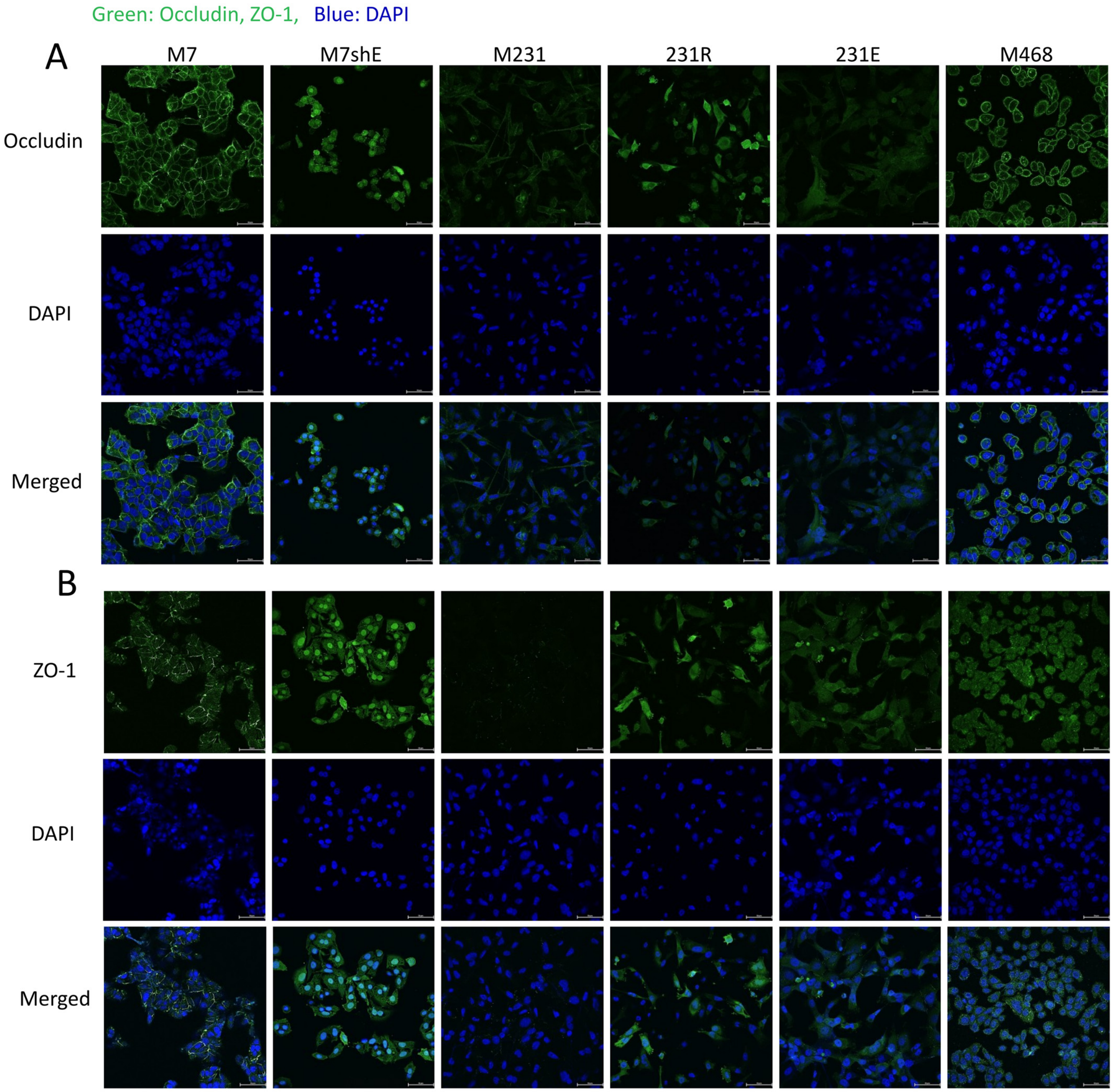

Supplementary Figure S3. Localization of Occludin and ZO-1 in different breast cancer cells using immunofluorescence staining. **(A)** Occludin, a tight junction protein was prominently expressed in M7 and M468 cells, however, was absent or internalized in M7shE and highly metastatic M231, 231R as well as 231E cells. **(B)** ZO-1, another tight junction marker was localized to cell-cell junction in M7 and M468 cells but was lost or internalized in M7shE, M231, 231R and 231E cells. Cell tight junctions were stained with either Occludin (green) or ZO-1 (green). Cell nucleus was counter-stained with DAPI (blue). Images were acquired at 400× magnification under Nikon A1 Confocal Microscope. Bars: 50 m.

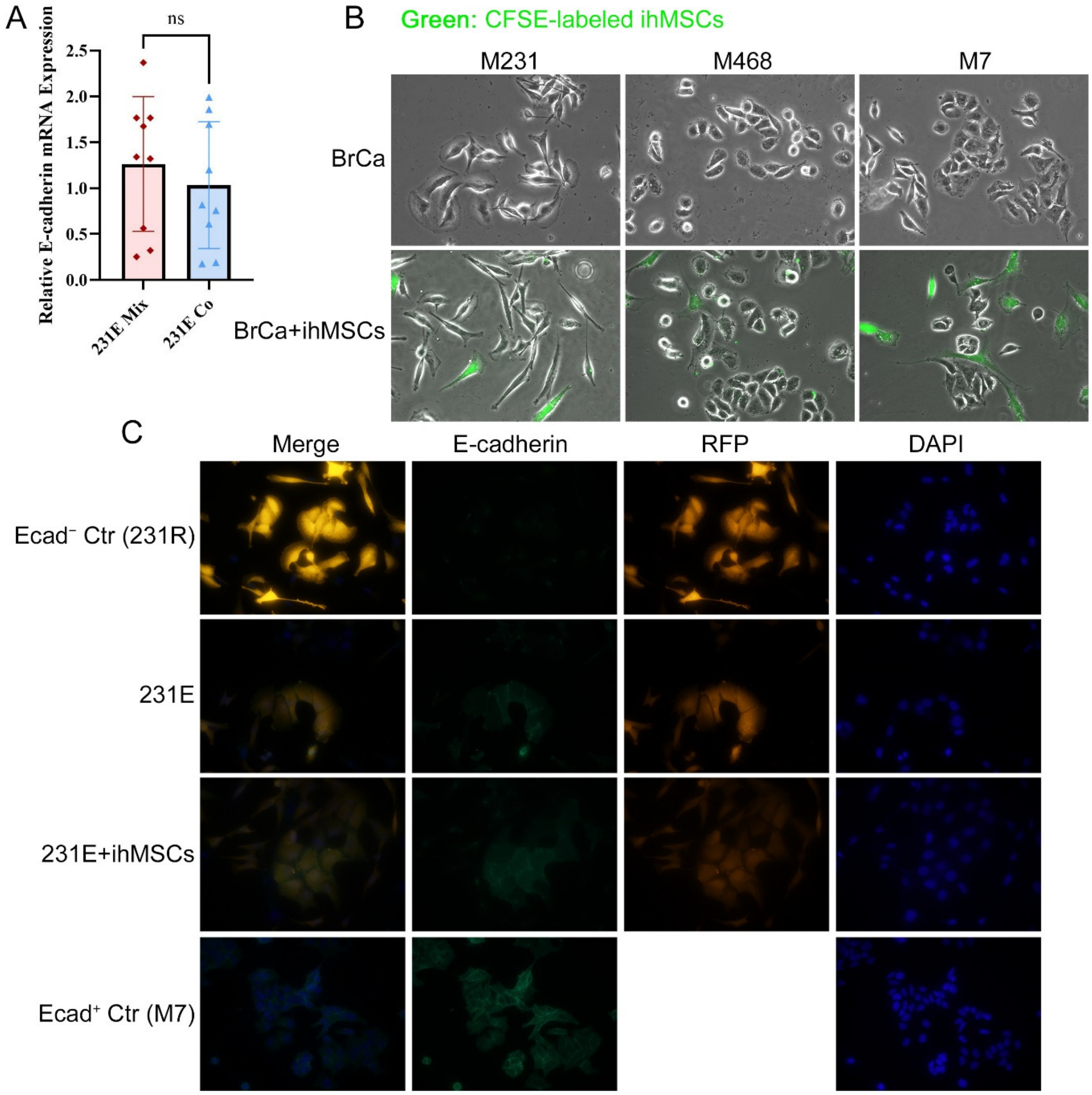

Supplementary Figure S4. Validation of immunoblotting results by RT-qPCR, morphological analysis, and immunofluorescence. **(A)** Relative E-cadherin mRNA expression in 231E cells under direct co-culture or monoculture conditions, measured by RT-qPCR and calculated using the $2^{-\Delta\Delta Ct}$ method (normalized to GAPDH and Ctr groups). **(B)** Representative images of M231, M468, and M7 cells cultured alone or directly co-cultured with carboxyfluorescein succinimidyl ester (CFSE)-labeled ihMSCs. ihMSCs were labeled with CFSE using CFSE Cell Proliferation Kit (Invitrogen, Cat# C34554) according to manufacturer's instructions prior to seeding and co-culturing with BrCa cells. Briefly, 500,000 ihMSCs were incubated in 1 mL PBS (without Ca^{2+} and Mg^{2+}) containing 5 μM CFSE at 37°C for 30 min. After adding 5 mL pre-warmed medium, cells were incubated for 5 min at room temperature, centrifuged, resuspended in fresh medium, and incubated for another 10 min. After 20 min of fixation with 3.7% formaldehyde and mounting with mounting buffer, images were captured under an Olympus IX70 inverted fluorescence microscope at 200× magnify. **(C)** Representative IF photos in 231E cells with or without ihMSC direct co-culture. Pictures were taken under an Olympus BX40 upright fluorescence microscope, using 400× magnify. $Ecad^{+}$ Ctr: E-cadherin-positive control. $Ecad^{-}$ Ctr: E-cadherin-negative control.

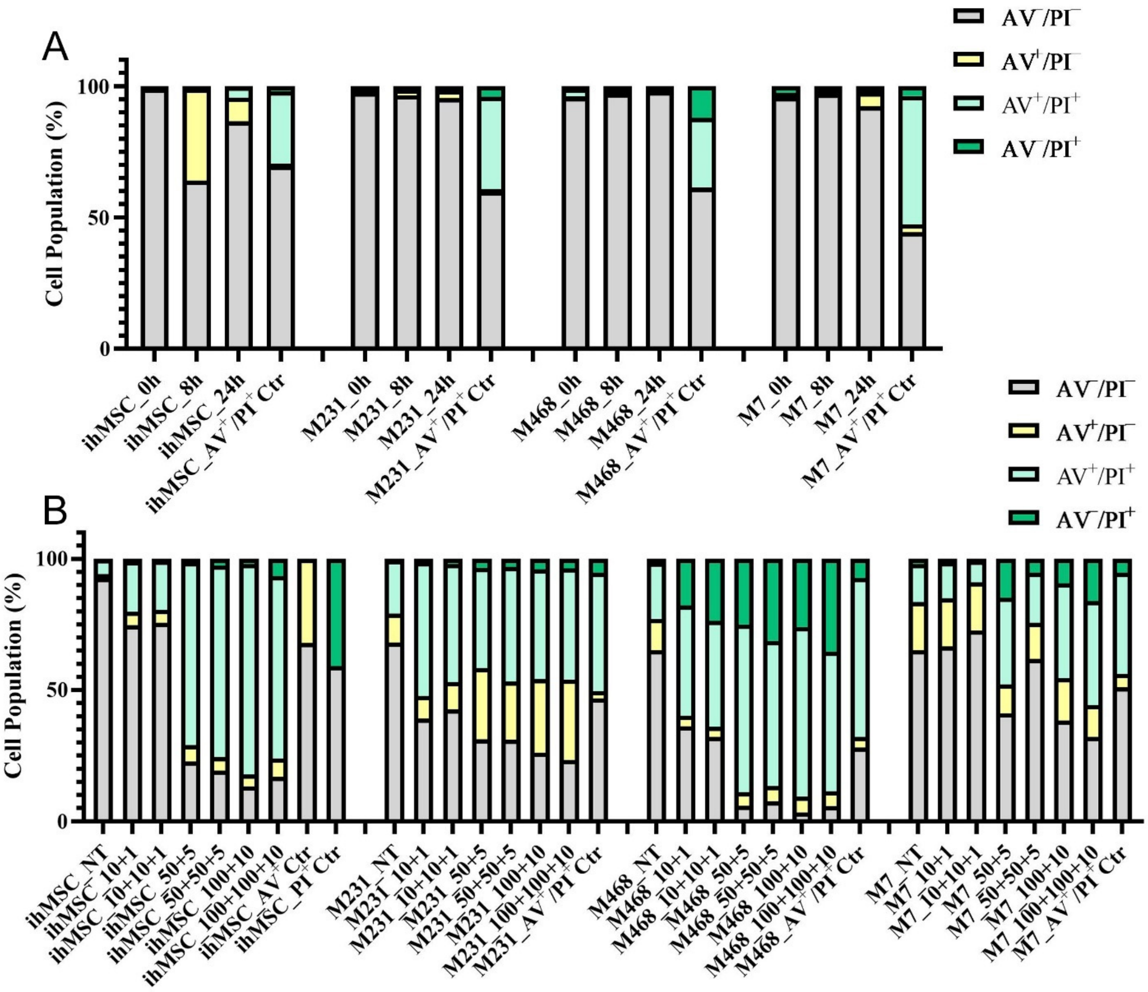

Supplementary Figure S5. Sensitivity of BrCa Cells and ihMSCs to FasL/TRAIL-Induced Apoptosis. **(A)** Flow cytometry analysis of AV/PI double staining in ihMSCs, M231, M468, and M7 cells treated with 100 ng/mL FasL (Thermo Fisher Scientific, Cat# 310-03H-10UG) and 20 μM CHX over a time course. Cells were cultured in RPMI-1640 supplemented with 10% FBS and 0.5% P/S until ~50% confluency, then switched to medium containing FasL/CHX with 0.5% FBS and 0.5% P/S. Both floating cells and adherent cells were collected by trypsinization and prepared for flow cytometry. **(B)** Flow cytometry analysis of AV/PI double staining in ihMSCs, M231, M468, and M7 cells treated with TRAIL, FasL, and CHX for 24 h under concentration gradients. Cells were cultured in RPMI-1640 with 10% FBS and 0.5% P/S until ~100% confluency, then treated with drug-containing RPMI-1640 supplemented with 0.5% FBS and 0.5% P/S. NT: non-treatment control. 10+1, 50+5, 100+10: TRAIL at 10, 50, or 100 ng/mL with CHX at 1, 5, or 10 μM. 10+10+1, 50+50+5, 100+100+10: TRAIL and FasL each at 10, 50, or 100 ng/mL with CHX at 1, 5, or 10 μM.

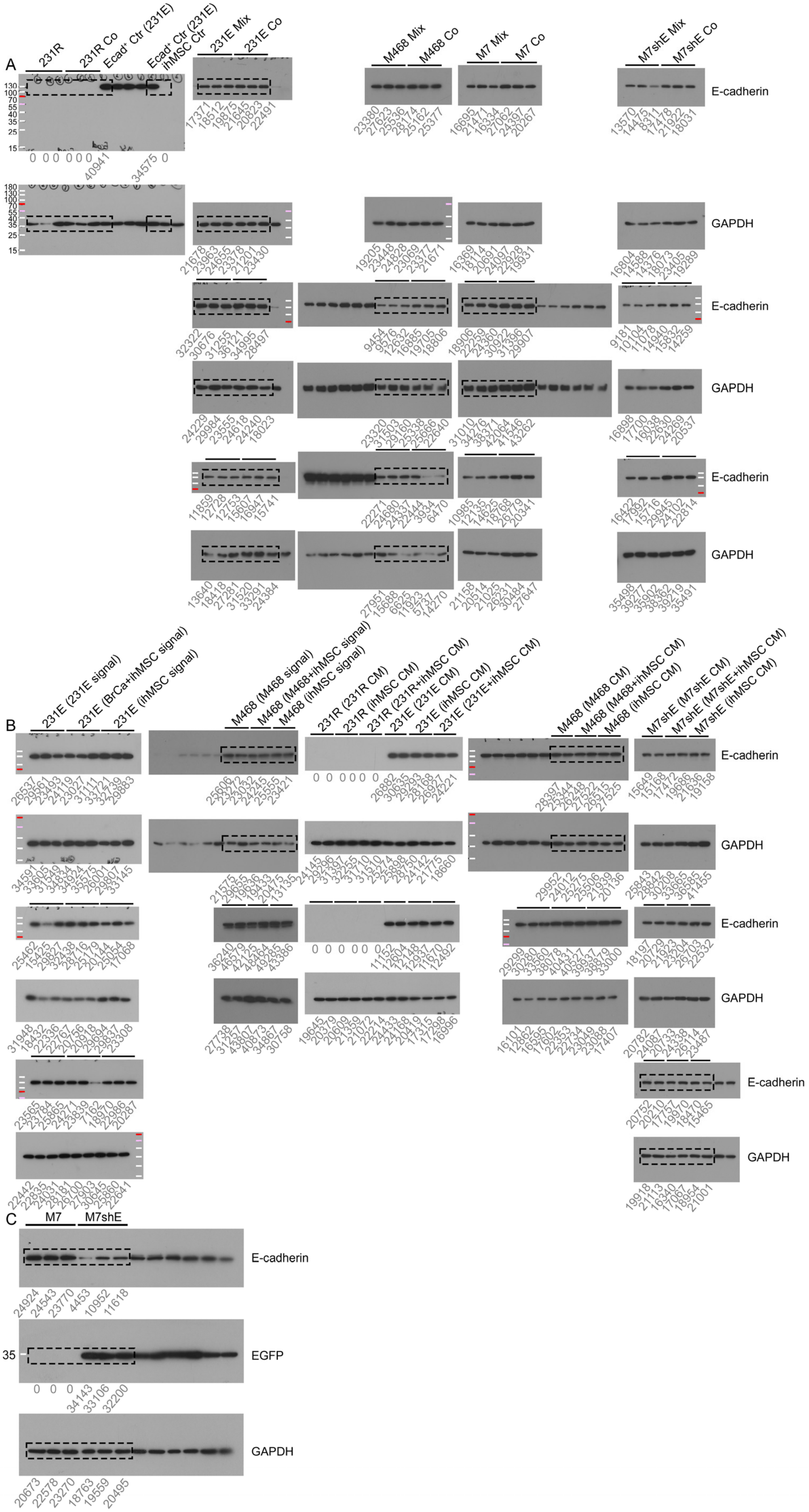
A
231R
231R Co
Ecad+ Ctr (231E)
Ecad+ Ctr (231E) ihMSC Ctr
231E Mix
231E Co
M468 Mix
M468 Co
M7 Mix
M7 Co
M7shE Mix
M7shE Co
E-cadherin
GAPDH
E-cadherin
GAPDH
E-cadherin
GAPDH
B
231E (231E signal)
231E (BrCa+ihMSC signal)
231E (ihMSC signal)
M468 (M468 signal)
M468 (M468+ihMSC signal)
M468 (ihMSC signal)
231R (231R CM)
231R (ihMSC CM)
231R (231R+ihMSC CM)
231E (231E CM)
231E (ihMSC CM)
231E (231E+ihMSC CM)
M468 (M468 CM)
M468 (M468+ihMSC CM)
M468 (ihMSC CM)
M7shE (M7shE CM)
M7shE (M7shE+ihMSC CM)
M7shE (ihMSC CM)
E-cadherin
GAPDH
E-cadherin
GAPDH
E-cadherin
GAPDH
C
M7
M7shE
E-cadherin
35
EGFP
GAPDH

Supplementary Figure S6. Uncropped WB images. **(A)** Full-length WB images showing E-cadherin and GAPDH expression in 231R, 231E, M468, M7, and M7shE cells cultured alone or in direct co-culture with ihMSCs. **(B)** Full-length WB images of E-cadherin and GAPDH expression in 231E and M468 cells exposed to BrCa signals, ihMSC signals, or BrCa+ihMSC signals in Transwell assays, and in 231R, 231E, M468, and M7shE cells treated with BrCa CM, ihMSC CM, or BrCa+ihMSC CM in CM experiments. These are from Figure 2C. **(C)** Full-length WB images showing E-cadherin, EGFP, and GAPDH expression in M7 and M7shE cells. Relevant densitometry measurements below the blots are provided. All blots are complete, as some of the membranes were cut before immunoblotting to differentially stain different size proteins. These are from Supplementary Figure S1.
